# Obturator hernia as a frequent finding during laparoscopic pelvic exploration: A retrospective observational study

**DOI:** 10.1097/MD.0000000000004102

**Published:** 2016-07-08

**Authors:** Sergio Susmallian, Oleg Ponomarenko, Royi Barnea, Haim Paran

**Affiliations:** a Department of Surgery, Assuta Medical Center, Tel Aviv; b Department of Chest Surgery, Assaf Harofeh Medical Center, Zerifin; c Department of Hemato-oncology, Assuta Medical Center, Tel Aviv; d Department of Surgery, Meir Medical Center, Kfar-Saba, Israel.

**Keywords:** incidence, inguinal hernia, obturator

## Abstract

Hernia through the obturator canal is usually unsuspected and hence undiagnosed. Patients with obturator hernias present as acute cases of intestinal obstruction secondary to strangulation or incarceration, with high rate of morbidity and mortality due to delayed diagnosis and treatment. The know incidence of obturator hernia is low, representing 0.073% (11 of 15,098) of all hernias repaired at the Mayo Clinic in a retrospective study of 15 years. In this study, we conducted a retrospective analysis of laparoscopic extraperitoneal hernia repairs that were performed between the years 2003 and 2007. All procedures were undertaken by 2 experienced surgeons who performed more than 150 previous surgeries. In 293 patients who underwent repair of bilateral or recurrent inguinal hernia, exploration of the obturator foramen was conducted looking for obturator hernia, which was found in 20 cases (6.82% of patients). The true incidence of obturator hernia is greater than that reported in the literature, and the chances of detecting hernia are greater if an equal number of men and women are scanned could be higher if pelvic scanning was performed.

## 1 Introduction

Hernia through the obturator canal is never externally visible, and rarely palpable, hence usually unsuspected and undiagnosed. This is accurately described in the work of Skandalakis regarding the obturator hernia.^[1]^

The obturator foramen is the largest foramen in the body, being formed by the rami of the ischium and pubis. The obturator canal is 2 to 3 cm long and 1 cm wide, and it contains the obturator nerve and vessels.^[2]^

Experience with hernia repair has taught us that obturator hernia occurs most often in old thin women, and classically presents as intestinal obstruction.

The known incidence of obturator hernia is low, representing 0.073% (11 of 15,098) of all hernias repaired at the Mayo Clinic in a retrospective study of 15 years. The incidence of obturator hernia in the Asian population is higher, representing about 1.6% of the population.^[3]^

Patients with obturator hernia present as acute cases of intestinal obstruction secondary to strangulation or incarceration, with the delay in diagnosis and treatment causing a high rate of morbidity and mortality. Advanced age of the patient and associated chronic diseases result in mortality as high as 47%.^[4]^

Researchers have postulated that obturator hernias begin with invagination of preperitoneal fat through the pelvic orifice of the obturator canal, forming a fat plug.^[5]^ From this point begins the formation of a true hernia forming the dimple and then the peritoneal hernia sac that may contain intestine giving symptoms of pain or obstruction.^[6]^

Development of the surgical technique of extraperitoneal repair for hernias facilitated dissection of the inguinal and pelvic areas, allowing for the recognition of types of hernia that previously went unnoticed.^[7]^

The aim of this study was to analyze the types of hernia encountered during laparoscopic extraperitoneal inguinal hernia repair, and to compare the observed incidence of obturator hernia to that reported in literature.

## 2 Methods

In this study, we conducted a retrospective analysis of laparoscopic extraperitoneal hernia repairs that were performed during the years 2003 to 2007, in a public hospital and elective patients only. All the patients that were selected for laparoscopic repair of bilateral groin hernia had bilateral inguinal hernia diagnosed by physical examination or recurrent inguinal hernia. Inguinoscrotal hernias were excluded. Obturator hernia was defined during laparoscopic approach as a wide obturator foramen that contains peritoneal sac (Fig. 2) or lipoma originated in the preperitoneal (Fig. 3) or paravesical fat tissue.

The study is an observational retrospective and thus ethical approval was not necessary.

Written informed consent was also not required for our study

### 2.1 Operative technique

Under general anesthesia and with the patient in the supine position, a preperitoneal plane was developed using a kidney-balloon trocar (Covidien-Medtronic, New Haven, CT). This plane was evaluated using the 45° scope. A structural trocar (Covidien-Medtronic) was then inserted and the space inflated with carbon dioxide at low pressure. One working 10 mm trocar was inserted in the midline as high as possible, and if necessary, another 5 mm trocar was inserted under the first one.

**Figure 1:**
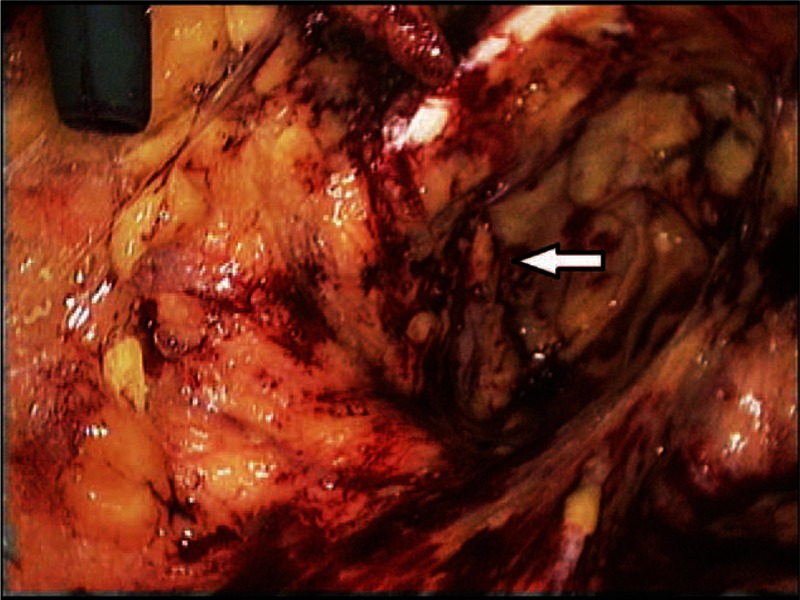
A photograph demonstrating left-sided normal obturator foramen.

Atraumatic dissection of the groin and pelvis was performed, the vessels (corona mortis, femoral, and epigastric) were recognized, then the cord was dissected and the peritoneal edge separated and retracted proximally wherever possible. We used the corona mortis vein as reference and followed it from the external iliac vein to reach the obturator foramen (Fig. 1). Broad dissection was performed laterally including the Bogros’ space, and superiorly releasing the peritoneum from the anterior abdominal wall.

### 2.2 Hernia repair

**Figure 2:**
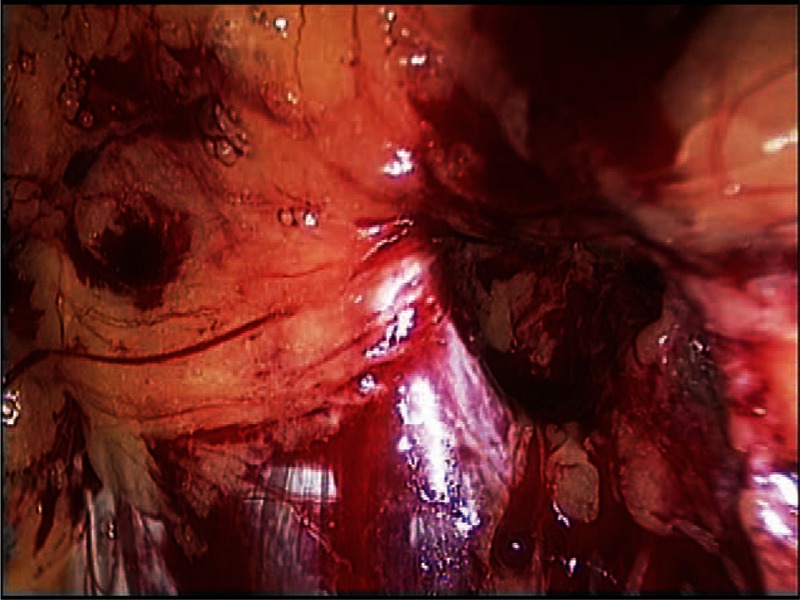
A photograph demonstrating right-sided obturator hernia.

**Figure 3:**
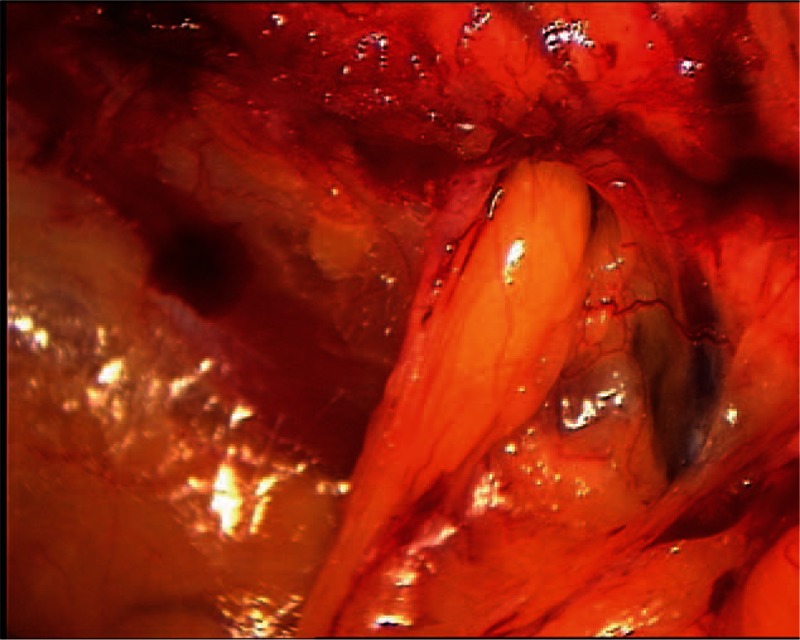
A photograph demonstrating lipoma reduction from right-sided obturator foramen.

To repair the groin we used a heavy poly-propylene mesh 15 cm × 15 cm, divided into 2 pieces, 15 cm × 5 cm and 10 cm × 5 cm. The 15 cm × 5 cm piece was inserted under the cord and fixed with helical tackers (Covidien-Medtronic) to the public ramus medially and to the transverse abdominal muscle laterally. The second 10 cm × 15 cm piece of mesh was placed above the cord overlapping with the first piece and fixed to the rectus abdominis and to the first mesh inserted. If obturator hernia was found repair was performed (Figs. 2 and 3).

**Figure 4:**
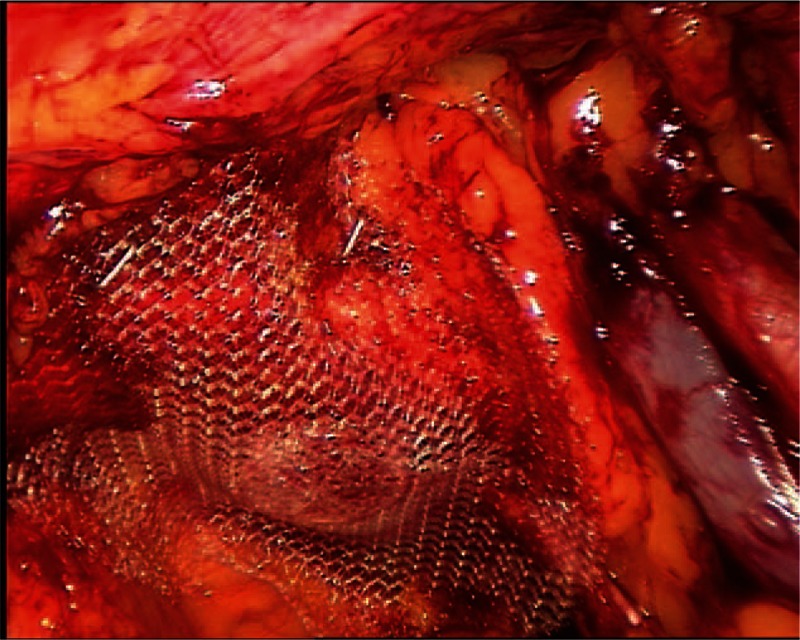
A photograph demonstrating repair of right-sided obturator hernia.

For obturator hernia repair, we used a circular poly-propylene mesh fixing it to the lacunar ligament of Gimbernat (Fig. 4).

The gas was released and bipivacaine was injected trough the trocar in the preperitoneal space. The patients were discharged from the hospital after 24 hours. A follow-up visit was scheduled 1 week after the surgery.

### 2.3 Statistical analysis

The results are presented as mean with range and as percentages where it is relevant.

## 3 Results

All procedures were performed by 2 experienced surgeons who have performed more than 150 previous surgeries.

Exploration of the obturator foramen was performed in 293 patients (96% men, 4% women) undergoing bilateral or recurrent inguinal hernia repair.

Obturator hernia was found in 20 cases (6.82%), 17 men and 3 women. The mean age was 53.8 years (range: 34–76 years; SD = 13.3). Of these 20 obturator hernias, 6 (30%) were unilateral, 3 on each side, and 14 (70%) were bilateral. In 19 cases fat pad was reduced from the foramen, and in 1 symptomatic female patient a peritoneal sac was reduced. The reason for operation was bilateral inguinal hernia in 18 patients (90%), and recurrent hernia in 2 patients (10%). The American Society of Anesthesia (ASA) score was 1 in 11 (55%) patients and 2 (45%) in 9 patients.

The average operative time was 44.5 minutes, and the average operative time for patients without obturator hernia was 45 minutes.

The median length of hospital stay was 1.1 days, 1 patient stayed for 3 days due to pain. The mean follow-up time was 15 months (range: 6–27 months), with no complications or recurrences.

## 4 Discussion

Hernia repair is one of the most common surgeries performed nowadays. According to H Kulacoglu, more than 20 million hernia repair surgeries are performed every year in the world and a large portion of them are inguinal.^[8]^

Obturator hernia is an infrequent but significant cause of intestinal obstruction. The incidence of obturator hernia is between 0.05% and 1.4% of all hernias.^[9]^

Although obturator hernia is considered as a rare type of hernia, its incidence in our patients was more than 6%. This incidence is much higher than that reported in literature.

Difficult diagnosis and subsequent delay in surgical treatment of acutely incarcerated obturator hernia can result in a mortality rate can be as high as 70%.^[9]^ The high mortality rate results from the patients’ advanced age as well as the delayed diagnosis in emergencies. Laparoscopic approach for the surgical repair of obturator hernia can contribute to a decreased mortality rate.

We agree with Shapiro that the total extraperitoneal approach during laparoscopic hernia repair is mandatory to explore all possible types of hernia, including obturator hernia.^[10]^

The routine search for a possible obturator hernia does not prolong operative time as 45 minutes was the average time for laparoscopic hernia repair and 44.5 minutes was the average time for the 20 obturator patients.

A large proportion (approximately two-thirds) of obturator hernias are not diagnosed until exploratory laparotomy.^[11]^

Furthermore, if obturator hernia is found, it must be repaired at the same session to avoid future complications and laparotomy.

Special consideration must be taken during laparoscopic exploration; proper recognition of the corona mortis vein and artery to prevent serious bleeding, and emphasizing the importance of fixing of the mesh to the Gimbernat and Cooper ligaments.^[12]^

The lifetime risk of inguinal hernia repair is high; at the current prevalence rates, we estimate it at 27% for men and 3% for women.^[13]^ In our study, we reported a male predominance in the incidence of inguinal hernia.

Obturator hernia affects women much more often than men (ratio 6–9:1).^[14]^ However, we reported a higher incidence of obturator hernia in men, in contrast to what is already known. A possible cause for this is the small sample size. Additionally, the small number of women operated upon in our series may have masked the true higher incidence of obturator hernia. The limitation of the study is to know the exactly occurrence of obturator hernia in patient having inguinal hernias from both genders. Women present higher incidence of obturator hernia than men. Since in our study group most of patients included are male, in order to know the true incidence of the obturator hernia, further studies must be conducted prospectively taking in mind the necessary equal numbers of male/female patients. Nevertheless, we believe that our findings are important as they show a higher obturator hernia incidence in our study group than the one that is reported in the literature.

## 5 Conclusion

The true incidence of obturator hernia is higher in patient with groin hernia than that reported in the literature for the general population and can be even higher if we perform scanning of the pelvis in the same number of men and women.

Based on our findings, during laparoscopic repair of inguinal hernia, the obturator foramen should be explored.

If obturator hernia is found, it should be repaired by fixing the mesh to the Gimbernat or Cooper ligaments.
